# Understanding resource utilization and mortality in COPD to support policy making: A microsimulation study

**DOI:** 10.1371/journal.pone.0236559

**Published:** 2020-08-20

**Authors:** Elizabeth G. Bond, Lusine Abrahamyan, Mohammad K. A. Khan, Andrea Gershon, Murray Krahn, Ping Li, Rajibul Mian, Nicholas Mitsakakis, Mohsen Sadatsafavi, Teresa To, Petros Pechlivanoglou, for the Canadian Respiratory Research Network

**Affiliations:** 1 Child Health Evaluative Sciences, The Hospital for Sick Children Research Institute, Toronto, ON, Canada; 2 Toronto General Hospital Research Institute, Toronto Health Economics and Technology Assessment (THETA) Collaborative, University of Toronto, Toronto, ON, Canada; 3 Institute of Health Policy, Management and Evaluation (IHPME), Dalla Lana School of Public Health, University of Toronto, Toronto, ON, Canada; 4 Dalla Lana School of Public Health, University of Toronto, Toronto, ON, Canada; 5 ICES, Toronto, ON, Canada; 6 Sunnybrook Research Institute, Toronto, ON, Canada; 7 University Health Network, Toronto General Hospital, Toronto, ON, Canada; 8 McMaster University, Hamilton, ON, Canada; 9 Collaboration for Outcomes Research and Evaluation, Faculty of Pharmaceutical Sciences, University of British Columbia, Vancouver, BC, Canada; Southern Cross University, UNITED STATES

## Abstract

Chronic obstructive pulmonary disease (COPD) poses a significant but heterogeneous burden to individuals and healthcare systems. Policymakers develop targeted policies to minimize this burden but need personalized tools to evaluate novel interventions and target them to subpopulations most likely to benefit. We developed a platform to identify subgroups that are at increased risk of emergency department visits, hospitalizations and mortality and to provide stratified patient input in economic evaluations of COPD interventions. We relied on administrative and survey data from Ontario, Canada and applied a combination of microsimulation and multi-state modeling methods. We illustrated the functionality of the platform by quantifying outcomes across smoking status (current, former, never smokers) and by estimating the effect of smoking cessation on resource use and survival, by comparing outcomes of hypothetical cohorts of smokers who quit at diagnosis and smokers that continued to smoke post diagnosis. The cumulative incidence of all-cause mortality was 37.9% (95% CI: 34.9, 41.4) for never smokers, 34.7% (95% CI: 32.1, 36.9) for current smokers, and 46.4% (95% CI: 43.6, 49.0) for former smokers, at 14 years. Over 14 years, smokers who did not quit at diagnosis had 16.3% (95% CI: 9.6, 38.4%) more COPD-related emergency department visits than smokers who quit at diagnosis. In summary, we combined methods from clinical and economic modeling to create a novel tool that policymakers and health economists can use to inform future COPD policy decisions and quantify the effect of modifying COPD risk factors on resource utilization and morality.

## Introduction

Chronic obstructive pulmonary disease (COPD) is the third leading cause of death worldwide and the fifth leading cause in Canada [1, 2]. Individuals with COPD have poorer quality of life, a higher comorbidity profile, and shorter life expectancy when compared to the general population [3, 4]. They are more frequent users of healthcare resources, accounting for at least one-fifth of all healthcare services used in Ontario [5]. In 2017, COPD exacerbations and acute bronchitis remained the leading causes of illness-related hospitalization in Canada [6]. In particular, in Ontario, individuals with COPD were responsible for 24% of hospital admissions and emergency department (ED) visits [5]. Individuals with COPD are also at a higher risk of ED readmission within 30 days (18.8%) [5]. The expected increase in COPD prevalence will continue to greatly impact this health service utilization in Ontario and in turn, will pose a significant financial challenge for provincial healthcare budgets [7].

Repeated COPD exacerbations have been shown to negatively affect both survival and quality of life, while also increasing healthcare costs [8–11]. As a result, prevention of COPD exacerbations has been identified as a key target of a number of large-scale policy interventions, worldwide [12–14]. Risk-factor stratification of individuals with COPD can increase efficient application of such interventions. A number of studies have attempted to identify risk factors of COPD exacerbations or hospitalizations and to develop prediction models to identify subpopulations at high risk of future exacerbations using administrative data [15–17].

Given the complex comorbidity profile of individuals with COPD, survival, hospitalizations and ED visits can vary and may be the result of either a COPD-related complication (e.g. exacerbations) or the result of a "competing" comorbid condition. Therefore, statistical models need to address the presence of competing risks, the frequency of transitions between states of care, and the recurrent event nature of the disease (e.g. multiple hospitalizations). In epidemiological and statistical literature, multi-state models are proposed as an extension of competing risk models that can address the complexities described above [18]. They can be combined with individual-level simulation (microsimulation) analysis to quantify the absolute and relative effects of time-dependent risk factors in a representative hypothetical population [19, 20]. Limited examples of such studies exist in the epidemiologic literature [21], and the combination of multi-state modeling and microsimulations has, to our knowledge, never been applied within a COPD context.

Policymakers commonly use economic evaluations to assess the long-term impact of implementing interventions. These evaluations most frequently rely on decision models in order to synthesize clinical and economic input from a variety of sources and to estimate the long-term effectiveness and cost-effectiveness of interventions [22, 23]. The personalized nature of COPD interventions poses a challenge in the development of such models, as they need to appropriately capture the individual heterogeneity in their structure and input parameters [24]. In addition, these models need to be validated against real-world data across different population strata to ensure that individual heterogeneity is accurately captured. The use of the combination of multi-state and microsimulation modeling in this study allows for subgroup-specific risk estimates that can be directly used either as input or as validation targets in such COPD policy models.

The aim of this study is to develop a platform that allows: 1) policymakers to identify subgroups of the COPD population that are at increased risk of ED visit, hospitalization and mortality and 2) health economists to obtain input or validation targets for personalized decision models in COPD. We achieved this through the estimation of a multi-state prediction model comprising six mutually exclusive states (1) In the community, post-COPD Diagnosis, (2) In the ED for a COPD-related reason, (3) In the ED for the non-COPD-related reason, (4) In the hospital for a COPD-related reason, (5) In the hospital for a non-COPD-related reason, (6) Dead from all causes) and the development of a microsimulation which allows for policy-specific subgroup estimations over long-term horizons.

## Methods

### Study design and setting

This is a retrospective cohort study of individuals diagnosed with COPD between January 1, 2001 and December 31, 2014. We used linked health administrative data that were collected in Ontario, Canada. In Ontario, healthcare resource use is routinely collected on all residents who participate in the single-payer healthcare system. The datasets were linked using unique encoded identifiers and analyzed at ICES in Toronto, Ontario, Canada.

### Data sources

Data were extracted from 11 health administrative databases including (1) Canadian Institute for Health Information-Discharge Abstract Database—diagnostic and discharge data, including the reasons for admission; (2) the National Ambulatory Care Reporting System—all ED visits in Ontario; and (3) Ontario Health Insurance Plan Claims Database—all physician claims data (S1 Table). The administrative data above were linked to responses from the Canadian Community Health Survey (CCHS), a survey that collects information on health status and determinants of health (e.g. smoking status) and is administered to a nationally representative sample [25, 26]. CCHS is administered in waves and the 2001, 2003–2005, 2007, 2009 and 2011 waves were used in this study.

### Study population and cohort

The study cohort was comprised of Ontario residents who completed at least one round of the CCHS survey and had a COPD diagnosis between January 1, 2001 and December 31, 2014. We identified COPD status using a previously validated case definition, specifically developed for health administrative data [27]. This definition captured newly diagnosed individuals that had one or more ambulatory claims and/or one or more hospitalizations for COPD and it has demonstrated a sensitivity of 85.0% and a specificity of 78.4% [27]. The administrative nature of the data allowed us to follow individuals with COPD throughout the entirety of their interactions with the Ontario healthcare system.

COPD-related and non-COPD-related ED visits and hospitalizations were extracted from the Institute of Clinical Evaluative Sciences data repository using International Statistical Classification of Diseases and Related Health Problems—9^th^ and 10^th^ revisions (ICD-9, ICD-10) codes (S2 Table) [27]. Individuals in the cohort were followed from their date of COPD diagnosis onward, until death, censoring, or end of follow-up. Individuals who had missing information for any of the covariates were excluded from the cohort. Individuals without at least one CCHS response were also excluded, and if more than one CCHS response was present, the most recent was used.

#### Ethics statement

The study protocol was approved by the research ethics board at The Hospital for Sick Children in Toronto, Ontario. The use of data in this project was authorized under section 45 of Ontario’s Personal Health Information Protection Act, which does not require review by a Research Ethics Board and allows ICES to receive and use health information without consent for purposes of analysis and compiling statistical information about the health care system of Ontario. The data used contained no personal identifiers and have been anonymized before being accessed by the study team.

### Outcomes

**Fig 1:**
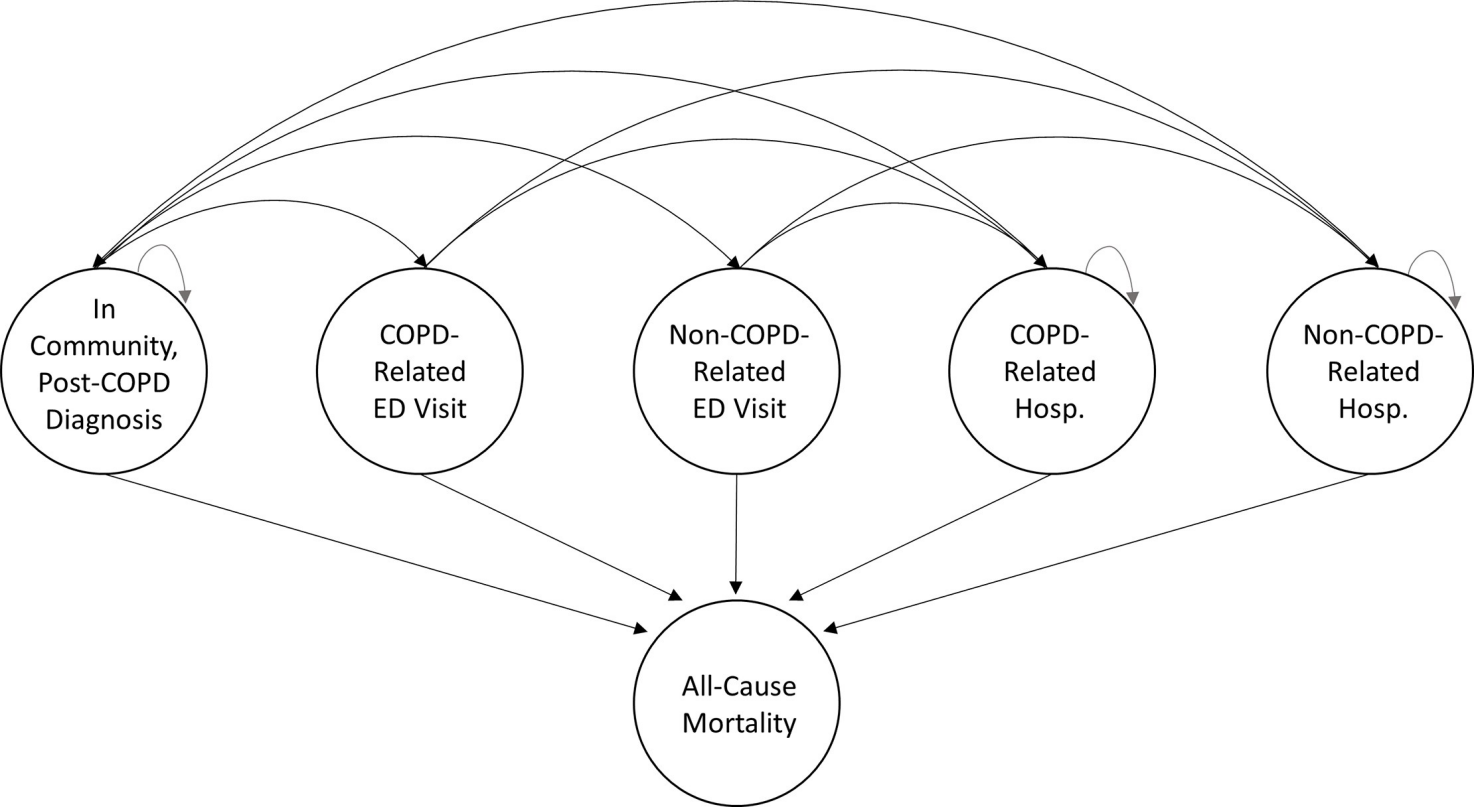
Model states and transitions of individuals with COPD. Six states: (1) In the community, post-COPD Diagnosis, (2) In the ED for a COPD-related reason, (3) In the ED for the non-COPD-related reason, (4) In the hospital for a COPD-related reason, (5) In the hospital for a non-COPD-related reason, (6) Dead from all causes. Abbreviations: COPD, Chronic Obstructive Pulmonary Disease; ED, Emergency Department; Hosp., Hospitalization.

Individuals with a COPD diagnosis were assumed to occupy one of the following six mutually exclusive states at any point in time: (1) In the community, post-COPD diagnosis; (2) COPD and (3) Non-COPD-related ED visit; (4) COPD and (5) Non-COPD-related hospitalization; (6) All-cause mortality (Fig 1). An event was classified as COPD-related based on ICD codes for both primary and secondary diagnoses [28] (S2 Table). COPD-related ED visits and hospitalizations were used as a proxy for severe COPD exacerbations [29]. Instead of COPD and non-COPD-related mortality, all-cause mortality was used because COPD is known to be underreported as a cause of death [30]. We constructed 17 possible direct transitions for individuals with COPD using the six mutually exclusive and/or competing states (Fig 1).

### Predictors

In order to gather relevant predictors, we conducted a thorough review of COPD literature [31–47]. Through this review, we deemed 18 predictors to be clinically and epidemiologically relevant to incorporate in the model of transitions between the states in our cohort (S3 Table). These predictors fell into three main categories, clinical, behavioral, and sociodemographic, with five of them being time-dependent (age and number of COPD-related and non-COPD-related ED visits and hospitalizations).

Smoking status, rurality and deprivation index were treated as categorical variables; age, ED visits, and hospitalizations were treated as continuous variables; and the remaining 10 predictors were treated as binary. Information regarding smoking history was collected from CCHS [26]. Smoking has been widely recognized as a main cause of COPD in high-income countries, and as such, it was important information for this cohort [48, 49]. Rurality and deprivation index were included to account for heterogeneity in healthcare access across the province. Additionally, we explored two other smoking-related covariates, second-hand smoking exposure and years smoked at diagnosis. Second-hand smoking information was limited within our cross-sectional survey data, without any historical exposure information, therefore it did not provide meaningful estimates. Years smoked at diagnosis was explored as an interaction with smoking history, but it did not improve our model fit. Finally, given the cross-sectional nature of the survey data and the temporality of smoking status, we fit a prediction model to impute smoking cessation for individuals that took the survey prior to their COPD diagnosis. This model was fit using individuals that had taken the survey after their COPD diagnosis, as this allowed us to observe the temporal effects of diagnosis on smoking.

### Statistical analysis

Our statistical analysis for the estimation of survival and resource use across different subgroups of the COPD population was comprised of two parts: 1) the estimation of multi-state and multinomial models and 2) the development of a microsimulation model.

#### Estimation

We used a combination of multi-state parametric regression modeling and multinomial logistic regression to estimate the daily transition probabilities between COPD states [18, 50–52]. Multi-state models are statistical models, rooted in survival analysis, which can accommodate the transition of individuals across multiple discrete states over time [18]. In the multi-state model, states are considered to be discrete, and can be either transient (e.g. hospitalization) or absorbing (e.g. death). Such models are an extension of competing risks methods that can also address the presence or recurrent entry to a given state e.g. multiple hospitalizations [53]. Transition among states is governed by transition intensities, which are defined as the instantaneous risk of moving from one state to another. Parametric multi-state models allow for time-dependent and non-proportional effects [54, 55]. Including multiple risk factors in the models allowed us to estimate their impact on each of the 17 transitions (Fig 1). The same covariates were used for each transition model. Time-dependent covariates (age, number of previous hospitalizations and ED visits) were incorporated in the models for the purpose of improving prediction accuracy [52]. Time-dependency is especially important for COPD as it allows for event history to be incorporated into the model [56, 57]. Each of our time-based transitions was modeled using six different distributions (Weibull, exponential, log-normal, log-logistic, gamma, and generalized gamma). We used multinomial regression with the same risk factors identified above, to estimate the probability of transition from ED to hospital, community, or death, as we assumed that transitions would occur within the same day of ED admission.

Model fit was assessed for all models using Akaike Information Criterion (AIC), Bayesian Information Criteria (BIC), and through visual assessment of survival curves [54, 58]. If the difference in AIC and BIC between distributions were negligible, we used the most parsimonious model.

#### Simulation and prediction

Covariate inference in such multi-state parametric models is typically hampered by two challenges: 1) the multiple transient states and recurrent events, and 2) the competing-risk nature of our data [59]. To address these challenges, we developed a microsimulation that was informed by the multi-state and multinomial model parameter estimates. This allowed us to quantify the absolute and relative parameter effects while also taking competing risks and recurrent events into consideration. Specifically, we implemented a Monte Carlo simulation approach similar to that described by Crowther and Lambert [54]. We incorporated both individual-level and parameter-level uncertainty using a two-level nested simulation. Below, we first describe the simulation and then apply the simulation to two different scenarios.

First, we generated a hypothetical synthetic cohort of individuals with a COPD diagnosis (n = 10,000) using baseline characteristics and their joint distributions from the real-world data. We then estimated absolute and relative effect measures I) across different cohort strata (stratified scenario) and II) in a counterfactual scenario. In the counterfactual scenario, the independent effect of a modifiable risk factor on outcomes can be achieved by rerunning the analysis with only the risk factor variable being modified in the original cohort, while all other characteristics remain the same.

Five events of interest (COPD-related and non-COPD-related ED visits and hospitalizations and all-cause mortality) were estimated using survival and cumulative event counts, in addition to length of stay (LOS). For cumulative or recurrent events, we employed an extension of the Mean Cumulative Count cohort study method by Dong et al., an extension of Nelson’s Mean Cumulative Function, which accounts for both the recurrent and competing risk nature of the events [60]. This approach allowed to report easily-interpretable mean event counts, instead of transition specific odd rations and hazard ratios. To propagate parameter-level uncertainty, the simulation was run 200 times using randomly sampled values from the multivariate distribution of each fitted model covariate set (S6 and S7 Tables). The outcomes of these simulations were used to generate and plot mean values and spline-based 95% empirical confidence intervals (CI) around the means were constructed. All analyses were completed using the statistical software R and several packages were used including “flexsurv”, “doParallel”, and “foreach” [52, 61–63].

#### Illustration of the approach

For the purposes of showcasing the functionality from the combined approach presented above, we investigated a stratified and a counterfactual scenario. Absolute and relative effect measures were estimated across (1) strata of current smokers, Former and never smokers and (2) Current smokers who continued smoking after diagnosis (non-quitters) and a counterfactual cohort of current smokers that quit smoking at diagnosis (quitters). Users of these models can easily estimate outcomes for any risk factors they wish, as we provide the annotated and easily reproducible R code and the parameter values for all modes in a GitHub repository (https://github.com/lizbond/COPD-SIM).

#### Validation

To ensure the validity of our analysis, we compared the model generated predictions to the extracted administrative data (internal validity) as well as to a different cohort of Canadians with COPD (external validity) [64]. Internal validation was conducted using the outcomes of survival probability and mean cumulative event counts, while external validation was conducted by comparing estimates of the average hospital LOS.

## Results

### Study cohort

Of the 811,477 Ontario residents diagnosed with COPD between 2001 and 2014, 19,372 completed at least one round of the CCHS survey. Our final analyses included 14,416 individuals (Table 1), as 4,956 of the survey respondents were excluded due to a missing smoking-related variable history (n = 4,433) or sociodemographic data (n = 523) (S1 Fig). The characteristics of the study cohort and those excluded were not appreciably different (S4 Table). During the study period, 0.6% (n = 83) of individuals were lost to follow-up and 19.5% (n = 2,804) of individuals died, leaving 79.9% (n = 11,529) of the cohort being alive at the end of follow-up. The mean length of observation was 5.9 years. From 2001 to 2014, there were 80,799 ED visits and 24,097 hospitalizations. Finally, just over half (n = 1,463) of the deaths occurred in-hospital, while only 5% (n = 158) of deaths occurred in the ED. A little more than half (55.1%) of our cohort was female, which was slightly higher than other population level studies performed in Ontario [65]. Similarly to another COPD, population study, in a different high-income country where 16.2% of individuals with COPD reported that they had never smoked, 17.2% of our cohort reported that they had never smoked [66]. Among the never smokers, 25% had a previous Asthma diagnosis (S5 Table). Interestingly, another large Canadian cohort study of individuals with COPD, where spirometry was used to identify individuals with COPD, found that 29% of individuals with COPD had never smoked [67]. Given differing COPD screening and identification methods and smoking definitions, one would expect some variation between these studies and estimates.

**Table 1:** Baseline characteristics of the individuals diagnosed with COPD, 2001–2014, Ontario, Canada (N = 14,416).

Characteristic	No. of Participants	%
**Age at Diagnosis, years**		
**Median (IQR)**	64	(54–74)
**Mean (SD)**	63.9	(13.8)
**Female**	7,953	55.2
**Deprivation Index**		
**1 (most advantaged)**	1,971	13.7
**2**	2,538	17.6
**3**	3,006	20.9
**4**	3,376	23.4
**5 (least advantaged)**	3,525	24.5
**Rurality Index of Ontario**		
**Urban**	6,681	46.3
**Suburban**	5,663	39.3
**Rural**	2,072	14.4
**Smoking Status**		
**Current**	6,331	43.9
**Former**	5,637	39.1
**Never**	2,448	17.0
**Comorbid Conditions**		
**Congestive Heart Failure**	1,588	11.0
**Ischemic Heart Disease**	2,527	17.5
**Cancer**	1,964	13.6
**Diabetes**	2,804	19.5
**Asthma**	2,758	19.1
**Dementia**	568	3.9
**Depression**	681	4.7
**Anxiety**	2,577	17.9
**Hypertension**	7,583	52.6
**Years of follow up**		
**Median (IQR)**	5	(2–9)
**Mean (SD)**	5.9	(3.9)
**Status in 2014**		
**Alive**	11,529	80.0
**Lost to follow-up**	83	0.6
**Death**	2,804	19.5
**No. COPD-Related ED Visits**		
**Median (IQR)**	0	(0–1)
**Mean (SD)**	0.7	(1.8)
**No. COPD-Related Hospitalizations**		
**Median (IQR)**	0	(0–1)
**Mean (SD)**	0.5	(1.2)

COPD, chronic obstructive pulmonary disease; ED, emergency department

### Multi-state model fit

Six distributional assumptions were assessed for fit and parsimony for each of the nine transitions in the multi-state model. The log-logistic distribution provided the best fit for five of the models (post-diagnosis to COPD-related ED visit, COPD-related hospitalization to post-diagnosis, COPD-related hospitalization to mortality, non-COPD-related hospitalization to post-diagnosis, and non-COPD-related hospitalization to mortality). The Weibull distribution provided the best fit for transitions to non-COPD-related ED visits and hospitalizations. Finally, the log-normal distribution provided the best fit post-diagnosis to COPD-related hospitalization and post-diagnosis to mortality.

### Scenario event prediction

**Fig 2:**
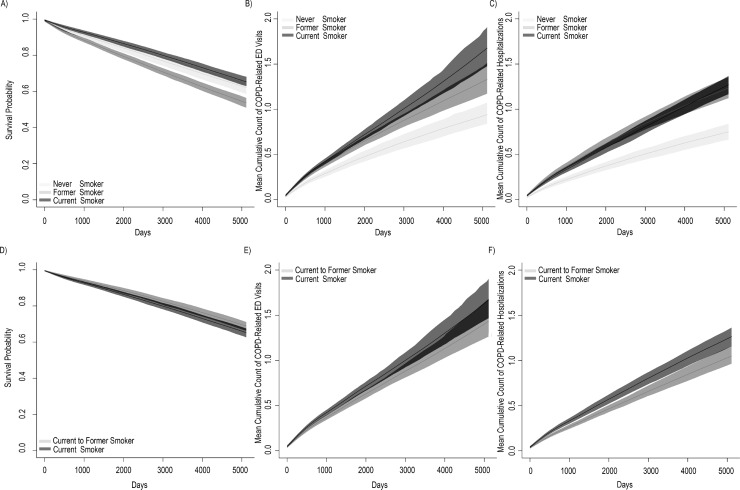
COPD microsimulation results. Predictions of all-cause mortality with 95% CI (left column), MCC with 95% CI of COPD-related ED visits (middle column), and MCC with 95% CI of COPD-related hospitalizations (right column). Current smokers vs. Former smokers vs. Never smokers (top row). Current smokers who continued smoking at diagnosis (non-quitters) vs. Current smokers who quit at diagnosis (quitters) (bottom row). Plots (A-C), Current smokers in dark gray, Former smokers in medium gray, and Never smokers in light gray; Plots (D-E), Current smokers [who continued smoking at diagnosis] in dark gray, Current to former smokers [smokers who quit at diagnosis] in medium gray. Abbreviations: CI, Confidence Interval; ED, Emergency Department; MCC, Mean Cumulative Count.

Scenario 1: The probability of survival was 65.3% (95% CI: 63.1, 67.9%) for current smokers, 53.6% (95% CI: 51.0, 56.4%) for former smokers and 62.1% (95% CI: 58.6, 65.1) for never smokers, at 14 years. This finding is likely the result of differences in comorbidity and age among three categories (S5 Table). The Mean Cumulative Count of COPD-related ED visits for current smokers remained higher than both former and never smokers, over the entirety of the simulated, 14 year observation period (Fig 2). At 14 years, current smokers have experienced, on average, 0.73 (95% CI: 0.64, 0.84) more COPD-related ED visits than never smokers, while former smokers have experienced 0.39 (95% CI: 0.33, 0.43) more COPD-related ED visits as never smokers.

Scenario 2: The probability of survival was 65.2% (95% CI: 62.7, 67.4) for non-quitters and 68.9% (95% CI: 66.3, 71.1) for quitters, at 14 years. Additionally, non-quitters averaged 16.4% (95% CI: 9.6, 38.4%) more COPD-related ED visits and 20.4% (95% CI: 6.1, 34.9%) more COPD-related hospitalizations than quitters, at 14 years.

#### Validation

Regarding internal validation, when stratifying both the real-world data and synthetic data by smoking history, the results were very similar, with each of the real-world survival probabilities falling within the 95% CI of the synthetic cohorts. For example, at 14 years, real-world, former smokers had a survival probability of 0.53, while the simulated former smokers had a survival probability of 0.54 (0.51, 0.56). Real-world, current smokers had a survival probability of 0.68, while the simulated current smokers had a survival probability of 0.65 (0.63, 0.68). Similarly, the predicted Mean Cumulative Count of COPD-related ED visits (0.64) was also very similar to the observed cohort (0.68). Regarding external validation, a 2010 report from the Canadian Thoracic Society noted that the average LOS for a COPD exacerbation was 10 days [64]. Similarly, our simulation demonstrated that the average COPD LOS, when discharged alive, was 10.17 days (95% CI: 10.15, 10.19). Computational barriers did not allow for cross-validation.

## Discussion

In this study, we developed a COPD microsimulation model that can be used to identify and quantify actionable risk factors and resource-intensive subgroups. Furthermore, the simulation allowed us to investigate and quantify the independent impact that specific covariates have on both resource use and survival. We quantified the impact of smoking and other risk factors on COPD-related and non-COPD-related ED visits and hospitalizations as well as all-cause mortality. Given the importance of reproducibility, the simulation was developed in such a way that it can be applied and replicated by both policy makers and researchers alike.

Methodologically, the simulation model demonstrates how multi-state modeling approaches can be applied in retrospective administrative data to identify risk factors for events of interest in the presence of competing risks, complex pathways and recurrent events. We used a parametric survival modeling approach that allows for extrapolation over a time horizon that extends over the data follow up. We used a microsimulation approach to generate meaningful estimates of covariate effects, similar to methods developed by Crowther and Lambert [54].

The results of this study can directly inform policy through identifying target actionable factors. For example, in our study, individuals who continued smoking at diagnosis averaged 40% more COPD-related ED visits, over 14 years. This finding reinforces the importance of policies and interventions dealing with smoking cessation programs that target newly diagnosed individuals. Additionally, the findings of this study can also indirectly affect decision making as they can serve as inputs in personalized, population-level simulation models in COPD. For example, reference policy models for COPD have recently been developed [68–70]. These models typically use estimates of COPD-related ED and hospitalization risk from prospective observational studies or clinical trials, which are based on relatively strict inclusion criteria and are at risk of protocol-driven disturbances in outcome measurement. The population-based nature of our data can make them an alternative choice for parameterizing such models. The multi-state and multinomial models from this analysis can directly be used as input parameters in models or can serve as calibration targets in the validation of the platform in an Ontario-based context. To facilitate reproducibility and reusability of this work we used R, an open-source language, and posted the simulation code and model estimates in a public repository on GitHub (https://github.com/lizbond/COPD-SIM). This allows other researchers to be able to reproduce the results and be able to estimate the absolute or relative effect of different combinations of covariates on the outcomes.

Our study is faced with a number of limitations. Regarding the available data, the information on some of the covariates of interest, such as smoking history, is cross-sectional. Additionally, because of the nature of survey data, as responses are self-reported, it is possible that smoking history of some individuals may have been misclassified. Due the administrative nature of the data, potentially informative clinical covariates, such as pulmonary function or FEV1, could not be included in the model [71]. However, history of hospitalization has been shown to be a proxy for exacerbations, a risk factor for future hospitalizations, declining lung function and mortality [15, 72–74]. Although we consulted experts in the field and completed a literature review, it is possible that we have missed ICD codes that may be considered COPD-related or relevant to a COPD-related event. Finally, while the narrow population in which the study was conducted may limit its generalizability, the work highlights a useful approach that is currently being tested in other populations. Future work examining how this approach may fare in other populations and jurisdictions may be warranted.

The methodology that we relied on provides powerful solutions that can possibly extend beyond what was utilized in this study (e.g. assuming interval censoring misclassification on the covariates or outcome). However, an important limitation, especially in the context of large administrative data and microsimulation, is the computational power that is required for such solutions to be implemented. Alternative approaches to simulation modeling such as the use of a discrete event simulation approach could possibly reduce computational needs [75]. Finally, the computational challenges in the estimation of the multi-state models reduced our ability to investigate model performance through time-dependent receiver operating characteristic curve and area under the curve or cross validation methods [76].

Despite the limitations, the models and subsequent simulation contribute to the progress of policy-level COPD predictions. Future plans for the simulation include further testing the validity and generalizability of our model, using external cohorts of individuals with COPD. We also plan to apply the simulation to cost prediction in this same Ontario-based COPD population. We will fit statistical models to individual-level administrative cost data, pooling together multiple sources of data from both inpatient and outpatient settings. Results from these cost models will then be integrated with the existing microsimulation in order to draw inference from individual-level predictors with regard to the economic burden of COPD.

## Supporting information

S1 Table Institute of clinical evaluative sciences databases. (DOCX)

S2 Table COPD-related international statistical classification of diseases and related health problems codes. (DOCX)

S3 Table COPD model predictors and covariates. (DOCX)

S4 Table Baseline characteristics of the individuals diagnosed with COPD, 2001–2014, Ontario, Canada. (DOCX)

S5 Table Age, sex and comorbidity by smoking status. (DOCX)

S6 Table Count of transitions between states. Abbreviations: DX, COPD Diagnosis and in the community; EDC, In the emergency department for a COPD-related reason; EDO, In the emergency department for the non-COPD-related reason; HC, In the hospital for a COPD-related reason; HO, In the hospital for a non-COPD-related reason; MO, All-cause mortality.
(DOCX)

S7 Table Multi-state model parameter estimates. Abbreviations: CHF, congestive heart failure; DX, in the community with a COPD Diagnosis; EDC, in the emergency department for a COPD-related reason; EDO, in the emergency department for the non-COPD-related reason; HC, in the hospital for a COPD-related reason; HO, in the hospital for a non-COPD-related reason; IHD, ischemic heart disease; MO, all-cause mortality.
(DOCX)

S8 Table Multinomial model parameter estimates. Abbreviations: CHF, congestive heart failure; DX, in the community with a COPD Diagnosis; EDC, in the emergency department for a COPD-related reason; EDO, in the emergency department for the non-COPD-related reason; HC, in the hospital for a COPD-related reason; HO, in the hospital for a non-COPD-related reason; IHD, ischemic heart disease; MO, all-cause mortality; No., number.
(DOCX)

S1 Fig Study cohort of individuals diagnosed with COPD, 2001–2014, Ontario, Canada. Abbreviations: CCHS, Canadian Community Health Survey; COPD, Chronic Obstructive Pulmonary Disease; ICES, Institute of Clinical Evaluative Sciences.
(TIF)

S2 Fig COPD microsimulation results. Absolute Difference with 95% CI, Current Smokers vs. Never Smokers in light gray and Former Smokers vs. Never Smokers in dark gray (top row) and Relative Difference with 95% CI, Current Smokers who continued smoking at diagnosis vs. Current smokers who quit at diagnosis (bottom row). Survival probability (left), COPD-related ED visits (middle), COPD-related hospitalizations (right). Abbreviations: CI, Confidence Interval; ED, Emergency Department.
(EPS)

## Abbreviations

AIC: Akaike Information Criterion
BIC: Bayesian Information Criteria
CCHS: Canadian Community Health Survey
CI: confidence interval
COPD: chronic obstructive pulmonary disease
ED: emergency department
LOS: length of stay
