# Supplementary material for: Understanding resource utilization and mortality in COPD to support policy making: A microsimulation study
Source: PLoS One. 2020 Aug 20;15(8):e0236559. doi: 10.1371/journal.pone.0236559 (PMC7444558; doi:10.1371/journal.pone.0236559)
Supplement: S1 Table — (DOCX) [file pone.0236559.s001.docx]

**Table S1. Institute of Clinical Evaluative Sciences Databases**

| **Name** | **Description** |
| --- | --- |
| COPD | Chronic Obstructive Pulmonary Disease |
| ASTHMA | Ontario Asthma dataset |
| CCHS | Canadian Community Health Survey |
| CHF | Congestive Heart Failure |
| ODD | Ontario Diabetes Dataset |
| OHIP | Ontario Health Insurance Plan Claims Database |
| RPBD | Registered Persons Database |
| IPDB | Institute of Clinical Evaluative Sciences Physician Database |
| ORGD | Vital Statistics - Deaths |
| HYPER | Ontario Hypertension dataset |
| OMHRS | Ontario Mental Health Reporting System |
| CIHI-DAD | Canadian Institute for Health - Discharge Abstract Database |
| CIHI-SDS | Canadian Institute for Health - Same Day Surgery Database |
| CIHI-NACRS | Canadian Institute for Health - National Ambulatory Care Reporting System |
| CIHI-CCRS | Canadian Institute for Health - Continuing Care Reporting System |
| ODB | Ontario Drug Benefit Claims |
| HCD | Home Care Database |
| CENSUS | Ontario Census Area Profiles |
| LHIN | Local Health Integration Network |
