# Supplementary material for: Understanding resource utilization and mortality in COPD to support policy making: A microsimulation study
Source: PLoS One. 2020 Aug 20;15(8):e0236559. doi: 10.1371/journal.pone.0236559 (PMC7444558; doi:10.1371/journal.pone.0236559)
Supplement: S2 Table — (DOCX) [file pone.0236559.s002.docx]

**Table S2. COPD-Related International Statistical Classification of Diseases and**

**Related Health Problems codes**

| ICD-9 | | ICD-10 | |
| --- | --- | --- | --- |
| Code | **Description** | **Code** | **Description** |
| 487 | Influenza with pneumonia | J10 | Influenza |
| 480 | Viral pneumonia | J11 | Influenza due to unidentified influenza virus |
| 481 | Pneumococcal pneumonia | J12 | Adenoviral pneumonia |
| 482 | Other bacterial pneumonia | J13 | Pneumonia due to Streptococcus pneumonia |
| 483 | Pneumonia due to other specified organism | J14 | Pneumonia due to Hemophilus influenza |
| 073.0 | Ornithosis | J15 | Bacterial pneumonia, not elsewhere classified |
| 115.15 | Infection by Histoplasma duboisii, pneumonia | J16 | Pneumonia due to other infectious organisms, not elsewhere classified |
| 115.95 | Histoplasmosis pneumonia | J17 | Pneumonia in diseases classified elsewhere |
| 484.7 | Pneumonia in other systemic mycoses | J18 | Pneumonia, unspecified organism |
| 484.8 | Pneumonia in other infectious diseases classified elsewhere | J20 | Acute bronchitis |
| 517.1 | Rheumatic pneumonia | J22 | Unspecified acute lower respiratory infection |
| 485 | Bronchopneumonia, unspecified organism | J40 | Bronchitis, not specified as acute or chronic |
| 514 | Pulmonary congest/hypostasis | J41 | Simple and mucopurulent chronic bronchitis |
| 486 | Pneumonia, unspecified organism | J42 | Unspecified chronic bronchitis |
| 466 | Acute bronchitis and bronchiolitis | J43 | Emphysema |
| 519.8 | Other diseases of respiratory system, not elsewhere classified | J44 | Other chronic obstructive pulmonary disease |
| 490 | Bronchitis, unspecified organism | J47 | Bronchiectasis |
| 491 | Chronic bronchitis |  |  |
| 492 | Emphysema |  |  |
| 493.21 | Chronic obstructive asthma with status asthmaticus |  |  |
| 493.22 | Chronic obstructive asthma with (acute) exacerbation |  |  |
| 493.20 | Chronic obstructive asthma, unspecified |  |  |
| 496 | Chronic airway obstruction, not elsewhere classified |  |  |
| 494.1 | Bronchiectasis with acute exacerbation |  |  |
| 494.0 | Bronchiectasis without acute exacerbation |  |  |
