# Supplementary material for: Understanding resource utilization and mortality in COPD to support policy making: A microsimulation study
Source: PLoS One. 2020 Aug 20;15(8):e0236559. doi: 10.1371/journal.pone.0236559 (PMC7444558; doi:10.1371/journal.pone.0236559)
Supplement: S3 Table — (DOCX) [file pone.0236559.s003.docx]

**Table S3. COPD Model Predictors and Covariates**

| Predictor | Definition | | Type | Reference |
| --- | --- | --- | --- | --- |
| Age | Continuous^b^ | Years | Sociodemographic | [[32](#_ENREF_32)]**,** [[31](#_ENREF_31)]**,** [[45](#_ENREF_45)]**,** [[46](#_ENREF_46)] |
| Sex | Binary | 0: Female  1: Male | Sociodemographic | [[35](#_ENREF_35)]**,** [[31](#_ENREF_31)] |
| Smoking history | Binary | 0: Never smoked a whole cigarette, non-smoker (Never smoker)  1: ever smoked at least a whole cigarette (Ever smoker) | Behavioral | [[36](#_ENREF_36)]**,** [[41](#_ENREF_41)]**,** [[31](#_ENREF_31)]**,** [[46](#_ENREF_46)] |
| Congestive Heart Failure | Binary | 0: No  1: Yes | Clinical | [[77](#_ENREF_77)]**,** [[78](#_ENREF_78)] |
| Ischemic Heart Disease | Binary | 0: No  1: Yes | Clinical | [[46](#_ENREF_46)]**,** [[77](#_ENREF_77)]**,** [[78](#_ENREF_78)] |
| Cancer | Binary | 0: No  1: Yes | Clinical | [[32](#_ENREF_32)]**,** [[46](#_ENREF_46)]**,** [[77](#_ENREF_77)] |
| Diabetes | Binary | 0: No  1: Yes | Clinical | [[32](#_ENREF_32)]**,** [[77](#_ENREF_77)]**,** [[78](#_ENREF_78)] |
| Asthma | Binary | 0: No  1: Yes | Clinical | [[39](#_ENREF_39)]**,** [[41](#_ENREF_41)]**,** [[46](#_ENREF_46)] |
| Dementia | Binary | 0: No  1: Yes | Clinical | [[79](#_ENREF_79)] |
| Depression | Binary | 0: No  1: Yes | Clinical | [[80](#_ENREF_80)]**,** [[81](#_ENREF_81)]**,** [[77](#_ENREF_77)]**,** [[78](#_ENREF_78)] |
| Anxiety | Binary | 0: No  1: Yes | Clinical | [[80](#_ENREF_80)]**,** [[78](#_ENREF_78)] |
| Hypertension | Binary | 0: No  1: Yes | Clinical | [[78](#_ENREF_78)] |
| Rurality | Categorical | Urban^a^  Suburban  Rural | Sociodemographic | [[82](#_ENREF_82)] |
| Deprivation | Categorical | 1: Most Advantaged^a^  2  3  4  5: Least Advantaged | Sociodemographic | [[34](#_ENREF_34)]**,** [[41](#_ENREF_41)]**,** [[46](#_ENREF_46)] |
| No. of COPD-related and non-COPD-related Emergency Department Visits | Continuous^b^ | Cumulative count | Clinical | [[83](#_ENREF_83)], [[84](#_ENREF_84)], [[5](#_ENREF_5)] |
| No. of COPD-related and non-COPD-related Hospitalizations | Continuous^b^ | Cumulative count | Clinical | [[83](#_ENREF_83)], [[84](#_ENREF_84)], [[5](#_ENREF_5)] |

^a^Reference Category

^b^Time dependent
