# Supplementary material for: Understanding resource utilization and mortality in COPD to support policy making: A microsimulation study
Source: PLoS One. 2020 Aug 20;15(8):e0236559. doi: 10.1371/journal.pone.0236559 (PMC7444558; doi:10.1371/journal.pone.0236559)
Supplement: S4 Table — (DOCX) [file pone.0236559.s004.docx]

**Table S4. Baseline Characteristics of the Individuals Diagnosed With COPD,**

**2001-2014, Ontario, Canada**

| Variable | All | Excluded | Analyzed |
| --- | --- | --- | --- |
|  | **No. (%)** | **No. (%)** | **No. (%)** |
|  | N=811,477 | N=797,061 | N=14,416 |
| Age at diagnosis |  |  |  |
| Mean (SD) | 61.90 (14.84) | 61.84 (14.85) | 63.85 (13.75) |
| Median (IQR) | 61 (50-74) | 61 (50-73) | 64 (54-74) |
| Age group |  |  |  |
| 35-49 | 194,380 (24.0) | 191,906 (24.1) | 2,474 (17.2) |
| 50-64 | 264,904 (32.6) | 260,099 (32.6) | 4,805 (33.3) |
| 65+ | 352,193 (43.4) | 345,056 (43.3) | 7,137 (49.5) |
| Status in 2014 |  |  |  |
| Alive | 600,449 (74.0) | 588,920 (73.9) | 11,529 (80.0) |
| Censor | 8,217 (1.0) | 8,134 (1.00) | 83 (0.6) |
| Death | 202,811 (25.0) | 200,007 (25.1) | 2,804 (19.5) |
| Female | 398,857 (49.2) | 390,904 (49) | 7953 (55.2) |
| Deprivation Index |  |  |  |
| 1 (most advantaged) | 130,893 (16.1) | 128,922 (16.2) | 1,971 (13.7) |
| 2 | 143,199 (17.6) | 140,661 (17.6) | 2,538 (17.6) |
| 3 | 157,124 (19.4) | 154,118 (19.3) | 3,006 (20.9) |
| 4 | 169,807 (20.9) | 166,431 (20.9) | 3,376 (23.4) |
| 5 (least advantaged) | 194,184 (23.9) | 190,659 (23.9) | 3,525 (24.5) |
| Missing | 16,270 (2.0) | 16,270 (2.00) | 0 |
| Rurality Index of Ontario |  |  |  |
| Urban (0-9) | 506,600 (62.4) | 499,919 (62.7) | 6,681 (46.3) |
| Suburban (10-44) | 232,431 (28.6) | 226,768 (28.5) | 5,663 (39.3) |
| Rural (45+) | 62,762 (7.7) | 60,690 (7.60) | 2,072 (14.4) |
| Missing | 9,684 (1.2) | 9,684 (1.20) |  |
| Smoking |  |  |  |
| No | 3,128 (0.4) | 680 (0.10) | 2,448 (17.0) |
| Yes | 15,186 (1.9) | 3,218 (0.40) | 11,968 (83.0) |
| N/A | 1,058 (0.1) | 1,058 (0.10) |  |
| Missing | 792,105 (97.6) | 792,105 (99.4) |  |
| Congestive Heart Failure | 92,615 (11.4) | 91,027 (11.4) | 1,588 (11.0) |
| Ischemic Heart Disease | 135,683 (16.7) | 133,156 (16.7) | 2,527 (17.5) |
| Cancer | 104,140 (12.8) | 102,176 (12.8) | 1,964 (13.6) |
| Diabetes | 154,475 (19.0) | 151,671 (19.0) | 2,804 (19.5) |
| Asthma | 163,103 (20.1) | 160,345 (20.1) | 2,758 (19.1) |
| Dementia | 45,604 (5.6) | 45,036 (5.70) | 568 (3.90) |
| Depression | 40,911 (5.0) | 40,230 (5.00) | 681 (4.70) |
| Anxiety | 160,365 (19.8) | 157,788 (19.8) | 2,577 (17.9) |
| Hypertension | 390,525 (48.1) | 382,942 (48.0) | 7,583 (52.6) |
| Rural |  |  |  |
| No | 682,011 (84.0) | 670,962 (84.2) | 11,049 (76.6) |
| Yes | 128,871 (15.9) | 125,504 (15.7) | 3,367 (23.4) |
| Missing | 595 (0.1) | 595 (0.10) | 0 |
