# Supplementary material for: Understanding resource utilization and mortality in COPD to support policy making: A microsimulation study
Source: PLoS One. 2020 Aug 20;15(8):e0236559. doi: 10.1371/journal.pone.0236559 (PMC7444558; doi:10.1371/journal.pone.0236559)
Supplement: S5 Table — (DOCX) [file pone.0236559.s005.docx]

**Table S5. Age, Sex and Comorbidity by Smoking Status**

| **Covariate** | **Current** | **Former** | **Never** |
| --- | --- | --- | --- |
| **Age at Diagnosis (mean)** | 59.3 | 68.2 | 70.6 |
| **Male** | 2,229 (44.3) | 3,093 (53.4) | 657 (26.9) |
| **Cardiovascular Disease** | 177 (3.5) | 248 (4.3) | 113 (4.6) |
| **Cancer** | 500 (9.9) | 943 (16.3) | 358 (14.6) |
| **Diabetes** | 739 (14.7) | 1,318 (22.8) | 510 (20.9) |
| **Asthma** | 800 (15.9) | 1,086 (18.8) | 642 (26.2) |

*Binary variables reports as N and % within respective smoking status
