# Supplementary material for: Understanding resource utilization and mortality in COPD to support policy making: A microsimulation study
Source: PLoS One. 2020 Aug 20;15(8):e0236559. doi: 10.1371/journal.pone.0236559 (PMC7444558; doi:10.1371/journal.pone.0236559)
Supplement: S6 Table — Abbreviations: DX, COPD Diagnosis and in the community; EDC, In the emergency department for a COPD-related reason; EDO, In the emergency department for the non-COPD-related reason; HC, In the hospital for a COPD-related reason; HO, In the hospital for a non-COPD-related reason; MO, All-cause mortality. (DOCX) [file pone.0236559.s006.docx]

**Table S6. Count of transitions between states**

| **Transitions** | | **To** | | | | | |
| --- | --- | --- | --- | --- | --- | --- | --- |
|  |  | **DX** | **EDC** | **EDO** | **HC** | **HO** | **MO** |
| **From** | **DX** | - | 9,930 | 70,869 | 1,608 | 5,799 | 1,183 |
|  | **EDC** | 6,443 | - | - | 3,115 | 352 | 16^a^ |
|  | **EDO** | 57,477 | - | - | 2,911 | 10,312 | 142^a^ |
|  | **HC** | 6,981 | - | - | - | - | 650 |
|  | **HO** | 15,644 | - | - | - | - | 813 |
| **Total** | | 86,545 | 9,930 | 70,869 | 7,634 | 16,463 | 2,804 |

^a^ < 200 total transitions

Abbreviations: DX, COPD Diagnosis and in the community; EDC, In the emergency department for a COPD-related reason; EDO, In the emergency department for the non-COPD-related reason; HC, In the hospital for a COPD-related reason; HO, In the hospital for a non-COPD-related reason; MO, All-cause mortality.
