# Supplementary material for: Understanding resource utilization and mortality in COPD to support policy making: A microsimulation study
Source: PLoS One. 2020 Aug 20;15(8):e0236559. doi: 10.1371/journal.pone.0236559 (PMC7444558; doi:10.1371/journal.pone.0236559)
Supplement: S7 Table — Abbreviations: CHF, congestive heart failure; DX, in the community with a COPD Diagnosis; EDC, in the emergency department for a COPD-related reason; EDO, in the emergency department for the non-COPD-related reason; HC, in the hospital for a COPD-related reason; HO, in the hospital for a non-COPD-related reason; IHD, ischemic heart disease; MO, all-cause mortality. (DOCX) [file pone.0236559.s007.docx]

**Table S7:** **Multi-state Model Parameter Estimates**

|  | | **Transition** | | | | | | | | |  |
| --- | --- | --- | --- | --- | --- | --- | --- | --- | --- | --- | --- |
|  |  | **DX to EDC** | **DX to EDO** | **DX to HC** | **DX to HO** | **DX to MO** | **HC to DX** | **HC to MO** | **HO to DX** | **HO to MO** |  |
|  |  | **Mean (SE)** | **Mean (SE)** | **Mean (SE)** | **Mean (SE)** | **Mean (SE)** | **Mean (SE)** | **Mean (SE)** | **Mean (SE)** | **Mean (SE)** |  |
| **Parameter** | Shape^a^ | 0.50 (0.00) | 0.52 (0.00) | 32.00 (1.10) | 0.45 (0.01) | 25.0 (0.71) | 2.00 (0.02) | 1.04 (0.03) | 2.00 (0.01) | 1.00 (0.03) |  |
|  | Scale^a^ | 280,000 (55,000) | 350.0 (20.0) | 10.00 (0.23) | 72,000 (17,000) | 4.60 (0.11) | 2.50 (0.24) | 3,100 (1,600) | 2.00 (0.11) | 2,500 (1,000) |  |
|  | Age | -0.01 (0.00) | 0.02 (0.00) | -0.02 (0.01) | 0.01 (0.00) | -0.12 (0.01) | 0.01 (0.00) | -0.04 (0.01) | 0.01 (0.00) | -0.03 (0.00) |  |
|  | Male | 0.02 (0.05) | 0.01 (0.02) | -0.59 (0.23) | -0.32 (0.06) | -0.27 (0.13) | -0.03 (0.02) | -0.11 (0.09) | -0.07 (0.02) | -0.16 (0.08) |  |
|  | Smoking None | Ref. | Ref. | Ref. | Ref. | Ref. | Ref. | Ref. | Ref. | Ref. |  |
|  | Smoking Former | -0.70 (0.07) | -0.01 (0.02) | -2.60 (0.37) | -0.05 (0.09) | -0.45 (0.17) | 0.02 (0.03) | -0.16 (0.14) | 0.01 (0.02) | -0.25 (0.11) |  |
|  | Smoking Current | -1.04 (0.08) | 0.13 (0.02) | -3.50 (0.39) | 0.14 (0.09) | -0.83 (0.19) | 0.04 (0.04) | -0.30 (0.16) | 0.08 (0.02) | -0.28 (0.13) |  |
|  | CHF Yes | -0.82 (0.07) | -0.46 (0.02) | -1.40 (0.32) | -0.18 (0.10) | -0.69 (0.16) | 0.12 (0.03) | 0.04 (0.11) | 0.05 (0.02) | -0.31 (0.10) |  |
|  | IHD Yes | -0.07 (0.06) | -0.21 (0.02) | -2.10 (0.26) | -0.25 (0.08) | -0.03 (0.15) | -0.03 (0.02) | -0.01 (0.10) | -0.02 (0.02) | 0.09 (0.09) |  |
|  | Cancer Yes | -0.12 (0.07) | -0.32 (0.02) | -2.80 (0.28) | -0.99 (0.08) | -1.19 (0.15) | 0.05 (0.03) | -0.29 (0.11) | 0.06 (0.02) | -0.26 (0.09) |  |
|  | Diabetes Yes | -0.15 (0.06) | -0.26 (0.02) | -0.95 (0.26) | -0.39 (0.08) | -0.12 (0.15) | 0.08 (0.02) | 0.07 (0.10) | 0.12 (0.02) | -0.10 (0.09) |  |
|  | Asthma Yes | -0.53 (0.06) | -0.16 (0.02) | -0.27 (0.28) | 0.04 (0.08) | 0.42 (0.17) | -0.02 (0.03) | 0.17 (0.13) | -0.06 (0.02) | 0.17 (0.12) |  |
|  | Dementia Yes | -0.56 (0.13) | -0.29 (0.04) | -1.90 (0.55) | -0.13 (0.18) | -2.10 (0.25) | 0.12 (0.05) | 0.21 (0.20) | 0.11 (0.04) | -0.12 (0.17) |  |
|  | Depression Yes | 0.23 (0.12) | 0.06 (0.04) | -0.24 (0.52) | 0.12 (0.16) | 0.32 (0.30) | 0.08 (0.05) | -0.14 (0.21) | -0.02 (0.03) | 0.25 (0.21) |  |
|  | Anxiety Yes | 0.03 (0.06) | -0.21 (0.02) | 0.26 (0.30) | 0.31 (0.08) | 0.17 (0.17) | -0.04 (0.03) | 0.04 (0.13) | -0.01 (0.02) | -0.01 (0.11) |  |
|  | Hypertension Yes | -0.22 (0.05) | -0.30 (0.02) | -1.50 (0.26) | -0.54 (0.07) | 0.08 (0.14) | -0.05 (0.02) | 0.02 (0.10) | 0.03 (0.02) | -0.04 (0.09) |  |
|  | Rurality Index Urban | Ref. | Ref. | Ref. | Ref. | Ref. | Ref. | Ref. | Ref. | Ref. |  |
|  | Rurality Index Suburban | -0.61 (0.05) | -0.22 (0.02) | -0.32 (0.25) | -0.22 (0.07) | 0.01 (0.13) | -0.06 (0.02) | -0.09 (0.10) | -0.05 (0.02) | -0.16 (0.09) |  |
|  | Rurality Index Rural | -0.93 (0.06) | -0.70 (0.02) | -0.93 (0.31) | -0.64 (0.09) | 0.98 (0.20) | -0.01 (0.03) | -0.04 (0.13) | -0.06 (0.02) | 0.20 (0.12) |  |
|  | Deprivation Index Q1 | Ref. | Ref. | Ref. | Ref. | Ref. | Ref. | Ref. | Ref. | Ref. |  |
|  | Deprivation Index Q2 | -0.09 (0.10) | -0.11 (0.03) | -0.34 (0.43) | 0.06 (0.11) | 0.06 (0.22) | 0.06 (0.04) | -0.15 (0.17) | -0.01 (0.03) | 0.08 (0.15) |  |
|  | Deprivation Index Q3 | -0.17 (0.09) | -0.22 (0.03) | 0.18 (0.41) | 0.12 (0.11) | 0.02 (0.21) | 0.01 (0.04) | -0.06 (0.17) | -0.01 (0.03) | 0.13 (0.14) |  |
|  | Deprivation Index Q4 | -0.32 (0.09) | -0.28 (0.03) | 0.26 (0.41) | 0.27 (0.11) | 0.16 (0.21) | 0.02 (0.04) | -0.19 (0.16) | 0.02 (0.03) | -0.04 (0.14) |  |
|  | Deprivation Index Q5 | -0.30 (0.09) | -0.42 (0.03) | -0.04 (0.39) | 0.18 (0.11) | -0.02 (0.21) | 0.03 (0.04) | -0.19 (0.16) | 0.05 (0.02) | -0.05 (0.14) |  |
|  | No. of EDC | -0.32 (0.01) | -0.04 (0.00) | 0.17 (0.08) | 0.18 (0.03) | 0.13 (0.04) | -0.04 (0.01) | 0.04 (0.02) | -0.02 (0.01) | -0.05 (0.03) |  |
|  | No. of HC | -0.21 (0.02) | -0.04 (0.01) | -1.10 (0.11) | -0.09 (0.04) | -0.62 (0.05) | 0.05 (0.01) | -0.05 (0.03) | 0.04 (0.01) | -0.02 (0.04) |  |
|  | No. of EDO | 0.02 (0.00) | -0.02 (0.00) | 0.34 (0.03) | 0.07 (0.01) | 0.06 (0.01) | 0.00 (0.00) | 0.01 (0.01) | 0.00 (0.00) | 0.01 (0.01) |  |
|  | No. of HO | 0.12 (0.02) | -0.08 (0.00) | 0.00 (0.08) | -0.30 (0.01) | -0.30 (0.03) | 0.01 (0.01) | -0.07 (0.02) | 0.01 (0.00) | -0.02 (0.02) |  |
|  | Distribution | Log logistic | Weibull | Log normal | Weibull | Log normal | Log logistic | Log logistic | Log logistic | Log logistic |  |

^a^ Shape and Scale for the Log normal distribution are the log of mean and standard deviation, respectively

Abbreviations: CHF, congestive heart failure; DX, in the community with a COPD Diagnosis; EDC, in the emergency department for a COPD-related reason; EDO, in the emergency department for the non-COPD-related reason; HC, in the hospital for a COPD-related reason; HO, in the hospital for a non-COPD-related reason; IHD, ischemic heart disease; MO, all-cause mortality.
