# Supplementary material for: Understanding resource utilization and mortality in COPD to support policy making: A microsimulation study
Source: PLoS One. 2020 Aug 20;15(8):e0236559. doi: 10.1371/journal.pone.0236559 (PMC7444558; doi:10.1371/journal.pone.0236559)
Supplement: S8 Table — Abbreviations: CHF, congestive heart failure; DX, in the community with a COPD Diagnosis; EDC, in the emergency department for a COPD-related reason; EDO, in the emergency department for the non-COPD-related reason; HC, in the hospital for a COPD-related reason; HO, in the hospital for a non-COPD-related reason; IHD, ischemic heart disease; MO, all-cause mortality; No., number. (DOCX) [file pone.0236559.s008.docx]

**Table S8:** **Multinomial Model Parameter Estimates**

|  | | **Transition Compared to Transition to DX** | | | | | |
| --- | --- | --- | --- | --- | --- | --- | --- |
|  |  | **EDC to HC** | **EDC to HO** | **EDC to MO** | **EDO to HC** | **EDO to HO** | **EDO to MO** |
|  |  | **Mean (SE)** | **Mean (SE)** | **Mean (SE)** | **Mean (SE)** | **Mean (SE)** | **Mean (SE)** |
| **Parameter** | Intercept | -3.50 (0.22) | -5.00 (0.52) | -8.60 (2.33) | -6.20 (0.18) | -3.60 (0.10) | -8.70 (0.77) |
|  | Age | 0.04 (0.00) | 0.04 (0.01) | 0.04 (0.03) | 0.04 (0.00) | 0.03 (0.00) | 0.03 (0.01) |
|  | Male | -0.04 (0.05) | 0.14 (0.13) | 0.45 (0.54) | 0.05 (0.04) | 0.07 (0.02) | 0.56 (0.19) |
|  | Smoking None | Ref. | Ref. | Ref. | Ref. | Ref. | Ref. |
|  | Smoking Former | 0.39 (0.08) | -0.22 (0.16) | 0.18 (0.84) | 0.45 (0.06) | 0.00 (0.03) | 0.15 (0.26) |
|  | Smoking Current | 0.61 (0.09) | -0.16 (0.18) | 0.68 (0.89) | 0.79 (0.07) | 0.11 (0.04) | 0.45 (0.29) |
|  | CHF Yes | 0.36 (0.07) | 0.43 (0.15) | 1.30 (0.60) | 0.56 (0.05) | 0.29 (0.03) | 0.40 (0.22) |
|  | IHD Yes | 0.00 (0.06) | 0.02 (0.14) | 0.07 (0.60) | 0.14 (0.05) | 0.11 (0.03) | 0.14 (0.20) |
|  | Cancer Yes | 0.09 (0.07) | 0.40 (0.15) | -0.27 (0.76) | 0.07 (0.05) | 0.06 (0.03) | 0.00 (0.23) |
|  | Diabetes Yes | 0.08 (0.06) | 0.20 (0.14) | 0.02 (0.61) | 0.26 (0.05) | 0.26 (0.03) | 0.12 (0.21) |
|  | Asthma Yes | -0.11 (0.06) | 0.07 (0.14) | 0.14 (0.62) | 0.10 (0.05) | -0.11 (0.03) | 0.07 (0.23) |
|  | Dementia Yes | 0.39 (0.14) | 0.78 (0.25) | -4.66 (0.00) | 0.38 (0.09) | 0.09 (0.06) | 0.67 (0.40) |
|  | Depression Yes | 0.18 (0.12) | -0.03 (0.29) | -4.10 (0.00) | -0.03 (0.10) | -0.01 (0.06) | -0.70 (0.56) |
|  | Anxiety Yes | -0.12 (0.07) | -0.11 (0.16) | 0.77 (0.60) | -0.12 (0.05) | -0.08 (0.03) | -0.31 (0.26) |
|  | Hypertension Yes | 0.12 (0.06) | 0.24 (0.14) | -0.06 (0.57) | 0.14 (0.05) | 0.15 (0.03) | 0.35 (0.21) |
|  | Rurality Index Urban | Ref. | Ref. | Ref. | Ref. | Ref. | Ref. |
|  | Rurality Index Suburban | -0.37 (0.06) | -0.61 (0.13) | 0.34 (0.56) | -0.21 (0.05) | -0.19 (0.03) | 0.17 (0.21) |
|  | Rurality Index Rural | -0.79 (0.07) | -1.11 (0.18) | -0.70 (0.91) | -0.44 (0.06) | -0.43 (0.03) | 0.24 (0.24) |
|  | Deprivation Index Q1 | Ref. | Ref. | Ref. | Ref. | Ref. | Ref. |
|  | Deprivation Index Q2 | -0.13 (0.10) | -0.17 (0.22) | -0.56 (0.82) | -0.06 (0.08) | -0.08 (0.05) | -0.42 (0.35) |
|  | Deprivation Index Q3 | -0.03 (0.10) | -0.29 (0.22) | -0.73 (0.82) | -0.10 (0.08) | -0.13 (0.04) | -0.17 (0.32) |
|  | Deprivation Index Q4 | 0.00 (0.09) | 0.01 (0.21) | -1.40 (0.91) | 0.02 (0.07) | -0.08 (0.04) | -0.07 (0.31) |
|  | Deprivation Index Q5 | -0.02 (0.09) | -0.06 (0.21) | -0.50 (0.75) | 0.07 (0.07) | -0.10 (0.04) | -0.26 (0.31) |
|  | No. of EDC | -0.12 (0.01) | -0.18 (0.04) | -0.11 (0.17) | -0.03 (0.01) | -0.08 (0.01) | 0.00 (0.05) |
|  | No. of HC | 0.34 (0.02) | 0.25 (0.05) | 0.29 (0.23) | 0.33 (0.02) | 0.12 (0.01) | 0.33 (0.07) |
|  | No. of EDO | -0.04 (0.00) | -0.02 (0.01) | -0.18 (0.10) | -0.07 (0.00) | -0.03 (0.00) | -0.11 (0.02) |
|  | No. of HO | 0.09 (0.02) | 0.16 (0.03) | 0.19 (0.23) | 0.07 (0.01) | 0.16 (0.01) | 0.19 (0.05) |

Abbreviations: CHF, congestive heart failure; DX, in the community with a COPD Diagnosis; EDC, in the emergency department for a COPD-related reason; EDO, in the emergency department for the non-COPD-related reason; HC, in the hospital for a COPD-related reason; HO, in the hospital for a non-COPD-related reason; IHD, ischemic heart disease; MO, all-cause mortality.
