# Supplementary figures and images for: Understanding resource utilization and mortality in COPD to support policy making: A microsimulation study
Source: PLoS One. 2020 Aug 20;15(8):e0236559. doi: 10.1371/journal.pone.0236559 (PMC7444558; doi:10.1371/journal.pone.0236559)

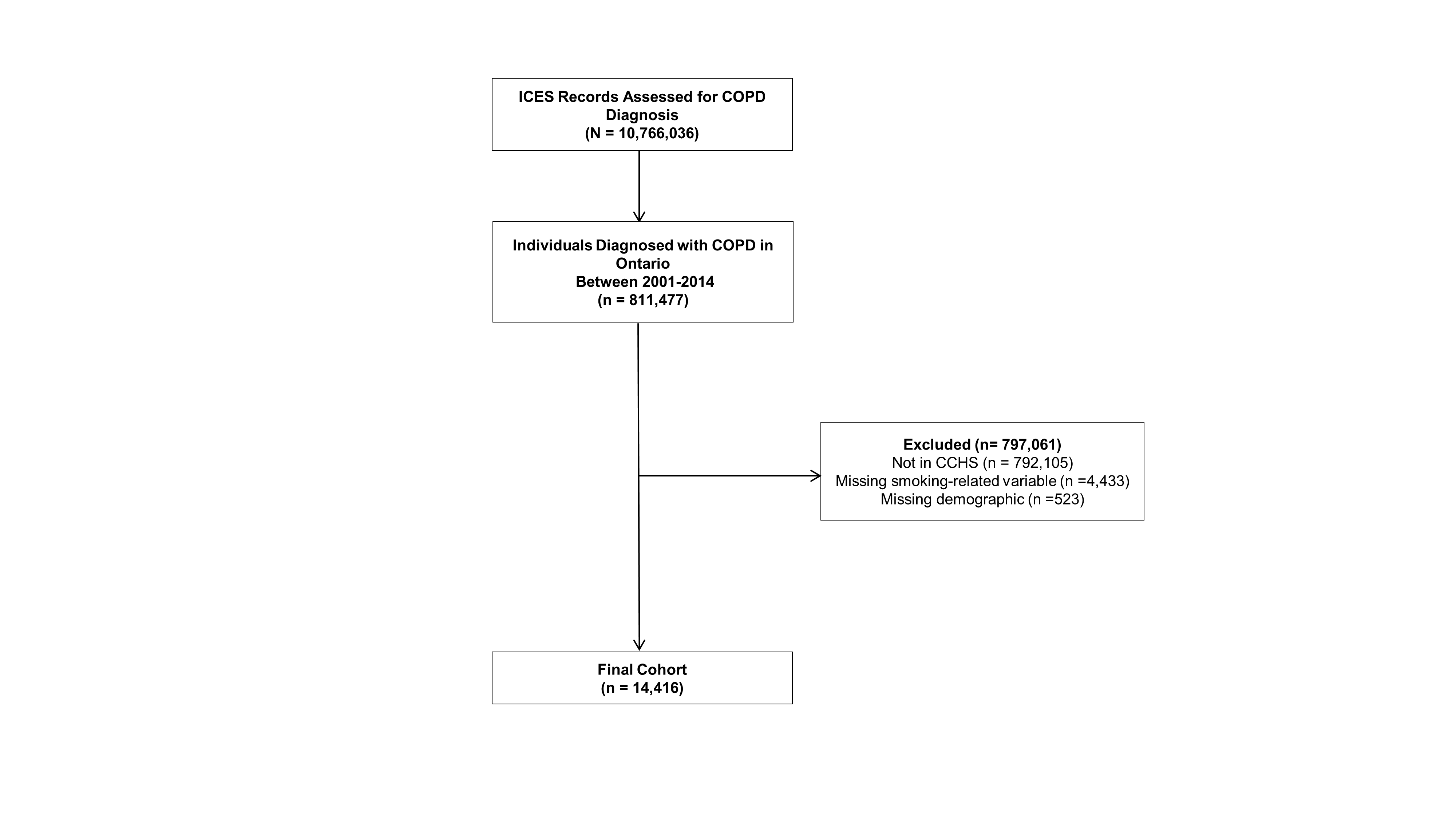

Supplement: S1 Fig — Abbreviations: CCHS, Canadian Community Health Survey; COPD, Chronic Obstructive Pulmonary Disease; ICES, Institute of Clinical Evaluative Sciences. (TIF) [file pone.0236559.s009.tif]
